# Fast ancestral gene order reconstruction of genomes with unequal gene content

**DOI:** 10.1186/s12859-016-1261-9

**Published:** 2016-11-11

**Authors:** Pedro Feijão, Eloi Araujo

**Affiliations:** 1 Technische Fakultät and CeBiTec, Universität Bielefeld, Universitätsstr. 25, Bielefeld, 33615 Germany; 2 Faculdade de Computação, Universidade Federal de Mato Grosso do Sul – UFMS, Campo Grande, MS Brazil

**Keywords:** Ancestral reconstruction, Small parsimony problem, Genome rearrangement, Double-cut-and-join, InDels, Gene insertions and deletions

## Abstract

**Background:**

During evolution, genomes are modified by large scale structural events, such as rearrangements, deletions or insertions of large blocks of DNA. Of particular interest, in order to better understand how this type of genomic evolution happens, is the reconstruction of ancestral genomes, given a phylogenetic tree with extant genomes at its leaves. One way of solving this problem is to assume a rearrangement model, such as Double Cut and Join (DCJ), and find a set of ancestral genomes that minimizes the number of events on the input tree. Since this problem is NP-hard for most rearrangement models, exact solutions are practical only for small instances, and heuristics have to be used for larger datasets. This type of approach can be called event-based. Another common approach is based on finding conserved structures between the input genomes, such as adjacencies between genes, possibly also assigning weights that indicate a measure of confidence or probability that this particular structure is present on each ancestral genome, and then finding a set of non conflicting adjacencies that optimize some given function, usually trying to maximize total weight and minimizing character changes in the tree. We call this type of methods homology-based.

**Results:**

In previous work, we proposed an ancestral reconstruction method that combines homology- and event-based ideas, using the concept of intermediate genomes, that arise in DCJ rearrangement scenarios. This method showed better rate of correctly reconstructed adjacencies than other methods, while also being faster, since the use of intermediate genomes greatly reduces the search space. Here, we generalize the intermediate genome concept to genomes with unequal gene content, extending our method to account for gene insertions and deletions of any length. In many of the simulated datasets, our proposed method had better results than MLGO and MGRA, two state-of-the-art algorithms for ancestral reconstruction with unequal gene content, while running much faster, making it more scalable to larger datasets.

**Conclusion:**

Studing ancestral reconstruction problems under a new light, using the concept of intermediate genomes, allows the design of very fast algorithms by greatly reducing the solution search space, while also giving very good results. The algorithms introduced in this paper were implemented in an open-source software called RINGO (ancestral Reconstruction with INtermediate GenOmes), available at https://github.com/pedrofeijao/RINGO.

## Background

With the increased availability of assembled genomes, methods that can analyse whole genome data and reconstruct phylogenetic trees based on large sctructural variations become increasingly relevant. A problem of great interest is the reconstruction of ancestral genomes based on gene order data. This is a classical problem in the field of genome rearrangements, where a large amount of research has been devoted, and still poses many challenges. In this problem, we are given a phylogenetic tree with extant genomes at its leaves, and need to reconstruct the gene orders at the internal nodes of the tree, corresponding to ancestral genomes.

We can broadly divide approaches of solving this problem in two categories. The first is a parsimonious approach, called *event*- or *distance-based*, were a rearrangement distance is given and the aim is to find ancestral genomes that minimize the length of the tree, defined as the total number of rearrangement events on all edges of the tree. Since BPAnalysis [1], the first proposed method, which was based the breakpoint distance, many other distance-based methods were developed, with different distances, such as the reversal distance (GRAPPA [2] and MGR [3]), the double cut and join (DCJ) distance [4, 5] (PATHGROUPS [6], GASTS [7] and MGRA [8, 9]), and the single cut or join (SCJ) distance [10] (SCJ Small Phylogeny [11]), just to cite a few examples.

Another category can be called *homology-based*, where methods usually do not apply rearrangement models directly, but instead treat conserved structures between the input genomes, such as conserved adjacencies or gene clusters, as binary characters (presence and absence). These characters can also have weights that represent a confidence or probability measure, and ancestral genomes are found by optimizing an objective function that might combine factors such as maximization of weights or probabilities, and minimizing character changes in the tree. Notable examples include the pioneer InferCARs [12], as well as GapAdj [13], ANGES [14], PMAG + [15, 16], ProCARs [17] and PhySca [18].

In our recent contribution to this field, we proposed a method that combines ideas from homology-based methods, namely adjacency weights, with the DCJ rearrangement model, by defining *intermediate genomes*, genomes that arise in optimal DCJ scenarios. We obtained promising results with this aproach, both in terms of running time and quality of the ancestral reconstruction [19].

Our previous approach, as well as most of the aforementioned methods (MGRA, GapAdj and PMAG + are exceptions), assume that all the input genomes have the same gene content, with just one copy of each gene, which is of course not a very realistic assumption, but it does make the problem much less complicated. In recent years, the focus has been shifted to include also gene content operations, such as gene insertion and deletions. MGRA and PMAG +, for instance, are updates of previous methods that dealt only with same gene content genomes.

In this direction, in this paper we extend the intermediate genome definition to unequal gene content genomes, by using the DCJ indel model [20]. Using this model, we study theoretical in “Preliminaries”, “Intermediate genomes” and “Ancestral reconstruction” sections and practical aspects in “Ancestral reconstruction algorithms” and “Results” sections. The complexity of the problem is unknown but we show that, depending on certain features of breakpoint graph we know how to solve the problem in polynomial time and in all other cases we have a FTP algorithms when we parameterize by the number *c* of the chromosomes. The ideas from this studying are partially used inspiring a description of a heuristic that has shown very good results regarding quality and time. In the last “Discussion” and “Conclusion” sections we discuss obtained results.

## Preliminaries

### Genes and genomes

A *gene* *g* is a sequence of two elements *g* ^*t*^ *g* ^*h*^ or *g* ^*h*^ *g* ^*t*^. So, *g* ^*t*^ *g* ^*h*^ and *g* ^*h*^ *g* ^*t*^ represent the same gene *g* with different orientation. We call *g* ^*h*^ and *g* ^*t*^ *extremities*, *g* ^*t*^ is a *tail* and *g* ^*h*^ is a *head* of *g*. Two different genes don’t share extremities. If $\mathcal {G}$ is a set of genes, denote $\mathcal {G}^{\pm } = \cup _{g \in \mathcal {G}} \{ g^{t}, g^{h} \}$. So, if $|\mathcal {G}| = n$, then $|\mathcal {G}^{\pm }|= 2n$.

A *chromosome* *C* is a sequence of genes that can be *linear* or *circular*. Denote by *V*_*C*_ the set of genes in *C*. If *C* is linear we represent it by adding a *telomere*, represented by the symbol ∘, at its endpoints. An *adjacency* in *C* is a pair *x* *y*≡*y* *x* such that *x* and *y* are in $V_{C}^{\pm } \cup \{ \circ \}$, implying that two genes are consecutive in *C*. If *x* or *y* is a telomere, this represents an extremity of a linear chromosome, and this type of adjacency is called a *telomeric adjacency*.

A *genome* is a set of chromosome and it is represented by the union of adjacency sets of their chromosomes. A genome is circular (linear) if all its chromosomes are circular (linear). For two genomes *A* and *B*, if *V*_*A*_=*V*_*B*_, we say that they have the *same gene content*. Conversely, if *V*_*A*_≠*V*_*B*_, they have *unequal gene content*.

### DCJ operation and the breakpoint graph

**Fig. 1:**
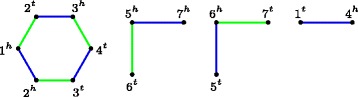
Breakpoint graph BP(*A,B*) of genomes *A*={∘1^*t*^,1^*h*^2^*t*^,2^*h*^3^*t*^,3^*h*^4^*t*^,4^*h*^∘,∘5^*t*^,5^*h*^6^*t*^,6^*h*^7^*t*^,7^*h*^∘} and *B*={1^*h*^2^*h*^,2^*t*^3^*h*^,3^*t*^4^*t*^,4^*h*^1^*t*^,∘6^*t*^,6^*h*^5^*t*^,5^*h*^7^*h*^,7^*t*^∘}. Edges of *A* are *green*, of *B* are *blue*

Let *A* be a genome, and *x* *y*≠*v* *w* two adjacencies in *A*. A *double cut and join operation* (DCJ) [4] on genome *A* is an operation that cuts two adjacencies of *A* and joins the free extremities in a different way. Many common rearrangement operations, like reversals and translocations, can be represented by a DCJ. Formally, a DCJ transforms *A* into genome *A*−{*x* *y,v* *w*}∪{*v* *y,x* *w*}. There is also the special case of *A*−{*x* *y*}∪{∘*x*,∘*y*} and the reverse case *A*−{∘*x*,∘*y*}∪{*x* *y*}, for *x,y*≠∘. For two genomes *A* and *B* with same gene content, the *DCJ distance* between *A* and *B* is the minimum number *d*_DCJ_(*A,B*) of DCJ operations that transforms *A* into *B*. The distance *d*_DCJ_(*A,B*) can be found with the *breakpoint graph* of *A* and *B*, denoted by BP(*A,B*), which is an edge-colored graph $G=(V_{A}^{\pm }, A \cup B)$, that is, the vertices are the gene extremities, and edges the adjacencies of both genomes (ignoring telomeric adjacencies). Edges from *A* have one color and edges from *B* have a different color. By definition, the breakpoint graph is collection of color alternating cycles and paths. Figure 1 shows and example of a breakpoint graph.

The DCJ distance is given by

1 $$\begin{array}{@{}rcl@{}} d_{\text{\texttt{DCJ}}} (A, B) & = & n - c(A, B) + \frac{p_{even}(A, B)}{2}, \end{array} $$

where $n=|\mathcal {G}|$ is the number of genes, *c*(*A,B*) and *p*_*even*_(*A,B*) are the number of cycles and the number of paths with even number of edges in BP(*A,B*) respectively, which can be found in linear time [5].

For genomes *A* and *B* with unequal gene content (*V*_*A*_≠*V*_*B*_), extra operations are required for inserting and deleting genes in *A* in order to transform *A* into *B*. Genes in *V*_*B*_−*V*_*A*_ are called *unique genes* of *B*, and conversely *V*_*A*_−*V*_*B*_ is the set of unique genes of *A*. An *insertion* in *A* consists in inserting a contiguous sequence of genes of *V*_*B*_−*V*_*A*_ in *A*, and a *deletion* in *A* is the inverse operation, i.e, removing a contiguous sequence of genes of *V*_*A*_−*V*_*B*_ from *A*. An *indel* is a general expression meaning an insertion or a deletion. The *DCJ-indel distance* between *A* and *B* is the minimum number of DCJs and indels required to transform *A* into *B*, and it is denoted as *d* DCJ ind(*A,B*). This distance can also be found in polynomial time, using two different approaches (Compeau [20] and Braga et al. [21]). Here, we use Compeau’s approach, which is based creating *prosthetic chromosomes* [22] in each genome, formed by the unique genes of the other, creating two new genomes with the same gene content.

### DCJ distance for unequal content genomes

For genomes *A* and *B* with unequal gene content, let $\mathcal {G} = V_{A} \cup V_{B}$ be the set of genes from both genomes. The breakpoint graph has a similar definition as before, changing only the vertex set, that is, $\text {\texttt {BP}}(A,B)=(\mathcal {G}^{\pm }, A \cup B)$, which means that new types of vertices and paths will be present.

**Fig. 2:**
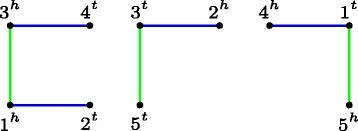
Breakpoint graph BP(*A,B*) of circular genomes *G*_*A*_=(1,−3,5) and *G*_*B*_=(1,2,3,4), with adjacency sets *A*={1^*h*^3^*h*^,3^*t*^5^*t*^,1^*t*^5^*h*^} and *B*={1^*h*^2^*t*^,2^*h*^3^*t*^,3^*h*^4^*t*^,4^*h*^1^*t*^}. Edges of *A* are *green*, and of *B* are *blue*. There is one *AA*-path and two *AB*-paths

A vertex *a* in BP(*A,B*) is *A*-open if $a \not \in V_{A}^{\pm }$, it is *B-open* if $a \not \in V_{B}^{\pm }$ and it is *not-open* otherwise. As well as telomeres, a missing gene in *A* or *B* appears as a endpoint of a path as we can see in Fig. 2. For a path *p* in BP(*A,B*), we say that *p* is *even* if the number of edges of *p* is even and it is *odd* otherwise; *p* is *not-open* if its endpoints are both not-open; *p* is an *AA-path* (*BB-path*) if its endpoints are both *A*-open (*B*-open); *p* is an *AB-path* if it has one *A*-open and one *B*-open endpoint; *p* is an *A-path* (*B-path*) if it has one *A*-open (*B*-open) and one not-open endpoint. Define *p*_*AB*_ as the number of *AB*-path and ${p_{A}^{o}}$ as the number of odd *A*-paths. Other notation for the number of odd/even-length paths (${p_{A}^{o}}$, ${p_{B}^{e}}$ and ${p_{B}^{o}}$) are defined analogously. When comparing two genomes *A* and *B*, a *singleton* is a circular chromosome *C* composed only by unique genes from one of the genomes, that is, *V*_*A*_∩*V*_*C*_=*∅* or *V*_*B*_∩*V*_*C*_=*∅*. The number of singletons for *A* and *B* is denoted by *s* *i* *n* *g*(*A,B*). Clearly, we can obtain *s* *i* *n* *g*(*A,B*) in polynomial time.

A *completion* for *A* and *B* is a pair of genomes *A* ^′^ and *B* ^′^ obtained from *A* and *B* by adding *artificial singletons* (prosthetic chromosomes) in *A* and *B* in such way the $V_{A'} = V_{B'} = \mathcal {G}$.

Compeau [20] showed that the DCJ-indel distance is given by

2 $$\begin{array}{@{}rcl@{}} d_{\text{\texttt{DCJ}}}^{\text{\texttt{ind}}} (A, B) = \min_{A', B'} \left\{ d_{\text{\texttt{DCJ}}} (A', B') \right\} + sing (A, B). \end{array} $$

A completion *A* ^′^ and *B* ^′^ for *A* and *B* such that minimize *d*_DCJ_(*A* ^′^,*B* ^′^) is called *optimal*.

**Fig. 3:**
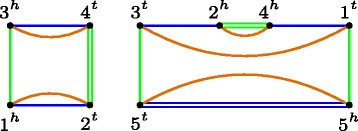
The unique optimal completion *C* of BP(*A,B*) from Fig. 2, where *A*-open (*B*-open) vertices are joined by *green* (*blue*) double edges, closing the *AA*-path and linking both *AB*-paths, which makes *d* DCJ ind(*A,B*)=*n*−*c*=3. The *orange* edges *M* ^′^={1^*h*^2^*t*^,2^*h*^4^*h*^, 4^*t*^3^*h*^,3^*t*^1^*t*^,5^*t*^5^*h*^} form a set of non-crossing chords covering all vertices of *C*. By Claim 2, *M* ^′^ leads to an intermediate genome. Notice that *S*={5^*t*^5^*h*^} is an artificial singleton, that is, a circular chromosome with only unique genes of *A*. Therefore, *M*=*M* ^′^−*S*={1^*h*^2^*t*^,2^*h*^4^*h*^,4^*t*^3^*h*^,3^*t*^1^*t*^}, representing the circular chromosome (1,2,−4,−3), is an intermediate genome. *M* is present in the optimal scenario $\mathcal {S}=\{M_{0}=A, M_{1}=(1,{-3})$, *M*_2_=(1,2,−4,−3),*M*_3_=*B*}, composed by one deletion, one insertion, and one reversal (DCJ)

In order to find optimal completions, consider the following definitions. For a set *A*, a *matching* *M* is a collection of disjoint subsets of *A*. *M* is a *perfect matching* of *A* or simply a perfect matching if the union of all sets in *M* is *A*. *M* is a *k-matching* if every set in *M* has *k* elements. A completion can then be seen as a perfect 2-matching of *A*-open vertices joined with a perfect 2-matching of *B*-open vertices in BP(*A,B*). In Fig. 3, we have an example of a breakpoint graph and a completion.

Let $\mathcal {C}$ be the set of all completions for *A* and *B*. If *n*_*A*_=|*V*_*B*_−*V*_*A*_| and *n*_*B*_=|*V*_*A*_−*V*_*B*_| are the number of unique genes in both genomes, then BP(*A,B*) has 2*n*_*A*_ *A*-open vertices and 2*n*_*B*_ *B*-open vertices. Since there are (2*n*_*A*_−1)!! different 2-matchings for the *A*-open vertices and (2*n*_*B*_−1)!! different 2-matchings for the *B*-open vertices, we have that

3 $$\begin{array}{@{}rcl@{}} |\mathcal{C}| & = &(2n_{A} - 1)!! \cdot (2n_{B} - 1)!!, \end{array} $$

which is exponential on the number of unique genes of *A* and *B*. However, an optimal completion can be found in polynomial time, which implies, since we can obtain *s* *i* *n* *g*(*A,B*) in polynomial time, that (2) can also be computed in polynomial time [20].

### Enumerating all optimal completions

The intuition behind finding an optimal completion is that Eq. (2) is minimized when the number of cycles and even paths of BP(*A,B*) is maximized. This guides the linking of components with *A*- and *B*-open vertices into creating as many cycles and even paths as possible. Therefore, *AA*-paths and *BB*-paths are always closed directly by linking their own *A*- or *B*-open vertices, since each becomes a cycle. *AB*-paths are usually linked in pairs, creating one cycle per pair. *A*-paths are also paired, ideally two paths with opposing parity, since this creates an even pair, and similarly for the *B*-paths. In many cases, this simple strategy is already enough to find optimal completions. Unfortunately, this can get more complicated when in some cases a triplet of components, specifically one *A*-path, one *AB*-path and one *B*-path can be linked in an optimal completion. In the following, we enumerate the space of all optimal completions, summarizing the results introduced by Compeau [20].

Let $\mathcal {C}^{*}$ be the space of all optimal completions for *A* and *B*. Using results from [20] we define a hypergraph $\mathcal {H}$ representing $\mathcal {C}^{*}$. The vertices represent components of the breakpoint graph, and hyperedges of $\mathcal {H}$ represent linked components that form a new component in a completion. In any completion, components without open vertices are not linked with other components. Also, AA-paths (BB-paths) become cycles by adding an edge between the two A-open (B-open) vertices in any optimal completion. Therefore, these components are not in $\mathcal {H}$.

In the following definitions, we use the notation of Cartesian product, but exclude pairs of identical elements, since a component can not be linked to itself. Let *V* be the set of vertices of $\mathcal {H}$. *V* is the union of the following sets, representing components of the BP(*A,B*): *Λ* ^*o*^, *Λ* ^*e*^, *Υ*, *Γ* ^*o*^ and *Γ* ^*e*^, the set of odd *A*-paths, even *A*-paths, *AB* paths, odd *B*-paths and even *B*-paths respectively. Consider the set of hyperedges of $\mathcal {H}$ that is the union of sets *T*_1_=*Λ* ^*o*^×*Λ* ^*e*^; *T*_2_=*Γ* ^*o*^×*Γ* ^*e*^; *T*_3_=*Υ*×*Υ*; *T*_4_=*Λ* ^*o*^×*Λ* ^*o*^; *T*_5_=*Λ* ^*e*^×*Λ* ^*e*^; *T*_6_=*Γ* ^*o*^×*Γ* ^*o*^; *T*_7_=*Γ* ^*e*^×*Γ* ^*e*^; *T*_8_=*Λ* ^*o*^×*Υ*×*Γ* ^*o*^; *T*_9_=*Λ* ^*o*^×*Υ*×*Γ* ^*e*^; *T*_10_=*Λ* ^*e*^×*Υ*×*Γ* ^*o*^; *T*_11_=*Λ* ^*e*^×*Υ*×*Γ* ^*e*^.

1. if *p*_*AB*_ is even, ${p_{A}^{o}} \le {p_{A}^{e}}$ and ${p_{B}^{o}} \ge {p_{B}^{e}}$, an optimal completion is any perfect matching using hyperedges in *T*_1_∪*T*_2_∪*T*_3_∪*T*_5_∪*T*_6_.
2. if *p*_*AB*_ is even, and ${p_{A}^{o}} \ge {p_{A}^{e}}$ and ${p_{B}^{o}} \le {p_{B}^{e}}$, an optimal completion is any perfect matching using hyperedges in *T*_1_∪*T*_2_∪*T*_3_∪*T*_4_∪*T*_7_.
3. if *p*_*AB*_ is odd, and ${p_{A}^{o}} \le {p_{A}^{e}}$ and ${p_{B}^{o}} \ge {p_{B}^{e}}$, an optimal completion is any perfect matching using only one hyperedge in *T*_10_ and hyperedges *T*_1_∪*T*_2_∪*T*_3_∪*T*_5_∪*T*_6_.
4. if *p*_*AB*_ is odd and ${p_{A}^{o}} \ge {p_{A}^{e}}$ and ${p_{B}^{o}} \le {p_{B}^{e}}$, an optimal completion is any perfect matching using only one hyperedge in *T*_9_ and hyperedges in *T*_1_∪*T*_2_∪*T*_3_∪*T*_4_∪*T*_7_.
5. if ${p_{A}^{o}} < {p_{A}^{e}}$ and ${p_{B}^{o}} < {p_{B}^{e}}$, an optimal completion is any perfect matching using hyperedges in *T*_1_∪*T*_2_∪*T*_3_∪*T*_5_∪*T*_7_∪*T*_11_.
6. if ${p_{A}^{o}} > {p_{A}^{e}}$ and ${p_{B}^{o}} > {p_{B}^{e}}$, an optimal completion is any perfect matching using hyperedges in *T*_1_∪*T*_2_∪*T*_3_∪*T*_4_∪*T*_6_∪*T*_8_.

#### **Claim 1**

Let $n = |\mathcal {G}|$ and *c* the sum of the number of chromosomes in *A* and *B*. Then, there are at most ((2*c*)!)^2^·*O*(*n* ^*c*^) different ways to choose a 3-matching in an optimal solution in $\mathcal {H}$.

#### *Proof*

Each set with three components represents one *A*-path, one *AB*-path and one *B*-path. Since each *A*-path and *B*-path has one telomere each and we have *c* chromosomes, there are *i*≤*c* triples in a solution. Considering that *i*=0,…,*c*, there are at most

$$ {n \choose 0} + {n \choose 1} + \ldots + {n \choose c} = O (n^{c}) $$

different ways to choose a set of *AB*-path to obtain triples in a optimal completion.

Once chosen a set of *AB*-path and we have to choose no more than 2*c* *A*-path and no more than 2*c* *B*-path. So, we have a total of no more than ((2*c*)!)^2^·*O*(*n* ^*c*^) different ways to choose a 3-matching in an optimal solution in $\mathcal {H}$. □

## Methods

In our previous approach, we used the concept of intermediate genomes to propose a new ancestral reconstruction method, in the context of genomes with same gene content [19]. We extend this approach here to genomes with unequal gene content, by dealing with gene insertion and deletion events.

In the following sections, every key aspect of the proposed method will be explained. Basic properties of intermediate genomes are described, based on existing results, and new properties for the case of genomes with unequal gene content are shown. Then, we show how the classic problems of small phylogeny and genome median can be reformulated adding intermediate genome constraints, also proposing a new problem, the *Maximum Weight Intermediate Genome*, that is at the core of our method.

Practical aspects such as estimating tree branch lengths and finding adjacency weights at each internal node of the tree are described. Finally, we describe the main algorithm, that iteratively reconstructs ancestors at internal nodes in a bottom-up approach, by using intermediate genome properties and adjacency weights.

## Intermediate genomes

In this section, we review some key combinatorial properties of intermediate genomes and extend the definition for genomes with unequal gene content, assuming that gene deletions and duplications have occurred.

### Basic properties of intermediate genomes

An *optimal DCJ scenario* between two genomes *A* and *B* is an ordered list of genomes $\mathcal {S} = (M_{0}, M_{1}, \dots, M_{k})$ where *k*=*d*_DCJ_(*A,B*), *A*=*M*_0_, *M*_*k*_=*B* and *M*_*i*_ can be obtained from *M*_*i*−1_ by applying a DCJ operation, for *i*=1,…,*k*. Any genome $M_{i} \in \mathcal {S}$ is called an *intermediate genome* of *A* and *B*.

Optimal DCJ scenarios can be found by dealing with each component in the breakpoint graph independently. A scenario that follows this strategy will be called *independent component scenario*. There are also optimal scenarios where a DCJ operations may act on two different components, specifically two even paths, but these are very rare [23]. Currently, we ignore recombination of even paths, in order to simplify the combinatorial analysis. In other context, a method was proposed to include this type of events [24], and we plan to add a similar extension to our framework as well.

Given breakpoint graph BP(*A,B*), a *circular breakpoint graph* can be obtained by transforming the paths into cycles as follows: i) to for each even path, add a new vertex ∘ and connect both extremities of the path to this new vertex; ii) for each odd path, add two new vertices ∘_1_ and ∘_2_ with and edge connecting both, and connect each extremity of the path to a different new vertex. This circular version of the breakpoint graph is composed only of cycles and it preserves the DCJ distance equation given by Eq. (1), adjusting *n* to *n*+*k*/2 to account for the extra number of *k* artificial vertices added [19].

The main property of intermediate genomes on independent component scenarios is given by the following theorem:

#### **Theorem 1** ([19])

Given genomes *A* and *B* with the same set of genes, a genome *M* is an intermediate genome of *A* and *B* in an independent component scenario if and only if the edges of *M* are non-crossing chords in the cycles of the circular BP(*A,B*), and *M* covers all vertices of BP(*A,B*).

In practice this makes it very easy to verify if a given genome is an intermediate genome, or even to create one given a choice of possible adjacencies, a key aspect of our ancestral reconstruction algorithm.

### Intermediate genomes for DCJ InDel scenarios

The definition of intermediate genomes for genomes with unequal content is the same as the original one, just considering optimal *DCJ-indel* scenarios, instead of DCJ only scenarios.

It is somewhat straightforward to extend the definition of intermediate genomes, using the DCJ-indel model of Compeau [20] and the concept of optimal completions. Given an optimal completion *C* of a breakpoint graph BP(*A,B*), we can create a *circular completion* by applying the operation of transforming all paths into cycles, similarly as done above to a breakpoint graph for genomes with the same gene content. After a circular completion is found, the resulting breakpoint graph is essentially the same as a breakpoint graph for genomes with same gene content. Therefore, we extend the results of Theorem 1 in the following claim.

#### **Claim 2**

Given genomes *A* and *B*, a circular optimal completion *C* of BP(*A,B*), and a set *M* ^′^ of non-crossing chords in the cycles of *C*, covering all vertices of *C*, the genome *M*=*M* ^′^−*S*, where *S* is the set of the adjacencies of all singletons of *M* ^′^ in respect to *A* and *B*, is an intermediate genome of *A* and *B*. Conversely, if *M* is an intermediate genome of *A* and *B*, there exists a circular optimal completion *C* of BP(*A,B*) and a set of adjacencies *S*, where *M* ^′^=*M*∪*S* is a set of non-crossing chords in the cycles of *C*, covering all of its vertices, and *S* forms the set of adjacencies of singletons of *M* ^′^ in respect to *A* and *B*.

Note that this result is general, also applicable for the same gene content genomes, since in this case we can consider that the breakpoint graph is directly an unique and optimal completion, and the set of singletons is always an empty set. Figures 2 and 3 show an example of an optimal completion and an intermediate genome.

## Ancestral reconstruction

In this section we explore how the concept of intermediate genomes can be used for ancestral reconstruction of gene orders.

In the context of rearrangement distance models, the ancestral reconstruction problem can be stated as: considering a measure distance *d*(*A,B*) between genomes *A* and *B*, given a tree *T* with *n* extant genomes at the leaves, find a labeling of the internal nodes corresponding to ancestral genomes, such that the total length of the tree, defined as the sum of all distances *d*(.) on the edges, is minimized. This is usually called the *small phylogeny problem*.

The simplest instance of this problem happens when only three genomes *A*, *B* and *C* are given, and we want to find a genome *M* minimizing *d*(*A,M*)+*d*(*B,M*)+*d*(*C,M*), the *genome median problem*. Despite being NP-hard for DCJ and many other models, it is well studied and many exact and heuristic methods have been proposed [25, 26],

Here we investigate new definitions of both the median problem and the small phylogeny problem that include intermediate genomes, motivated by the fact that some studies show that purely minimizing the tree length (or finding median genomes) might not be the best option for ancestral reconstruction [27].

Let IG(*A,B*) represent the set of intermediate genomes between *A* and *B*. For the median problem, we can use the fact that *d*(*A,M*)+*d*(*B,M*)=*d*(*A,B*) if *M* is in IG(*A,B*) to give the following definition.

### **Problem 1**

Given two genomes *A* and *B*, and an outgroup genome *C*, find an *M*∈IG(*A,B*) minimizing *d*(*C,M*).

### **Problem 2**

Given a rooted binary tree *T* with *n* extant genomes at the leaves, find a labeling of the internal nodes such that the tree length is minimized, and each genome on an internal node is an intermediate genome of its children.

### **Theorem 2**

The DCJ Intermediate Genome Median is NP-hard.

### *Proof*

A *balanced bicoloured graph* *G* is a graph where each vertex has the same number of red and blue incident edges, all vertices have degree two or four, and there is no cycle formed by edges of the same colour. An *alternating cycle* in *G* is a cycle where red and blue edges are alternating. The *breakpoint graph decomposition problem* (BGD) is to find a maximum number of edge-disjoint alternating cycles of *G*. This problem is NP-hard [28].

**Fig. 4:**
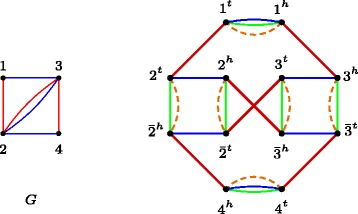
Given a balanced bicoloured graph *G* (at *left*), a breakpoint graph is constructed (at *right*), with genomes $A = \left \{ 1^{t} 1^{h}, 2^{t} 2^{h}, \bar {2}^{t} \bar {2}^{h}, 3^{t} 3^{h}, \bar {3}^{t} \bar {3}^{h}, 4^{t} 4^{h} \right \}$ (in *blue*), $B = \left \{ 1^{t} 1^{h}, 2^{t} \bar {2}^{h}, \bar {2}^{t} 2^{h}, 3^{t} \bar {3}^{h}, \bar {3}^{t} 3^{h}, 4^{t} 4^{h}\right \}$ (in *green*) and $C = \left \{ 1^{t} 2^{t}, \bar {2}^{t} 3^{t}, \bar {3}^{t} 4^{t}, 1^{h} 3^{h}, 2^{h} \bar {3}^{h}, \bar {2}^{h} 4^{h}\right \}$ (in *red*). In this example, *M*=*B*⊆*A*∪*B* is a median (in *dashed orange edges*)

A proof for this theorem can be derived directly from the original proof of NP-hardness of the DCJ median problem, where a reduction from BGD is performed [29]. In that proof, from an instance of the BGD with $G=(\mathcal {V}, \mathcal {B} \cup \mathcal {R})$, where $\mathcal {V}$ is a set of vertices and $\mathcal {B}$ and $\mathcal {R}$ are sets of blue and red edges, the genomes *A*, *B* and *C* on $\mathcal {G}$ are constructed. The set $\mathcal {G}$ contains one gene *X* for each degree 2 vertex and two genes *X* and $\bar {X}$ for each degree 4 vertex *X* of *G*. The set of adjacencies of *A* is $\left \{ X^{h} X^{t} : X \in \mathcal {G} \right \}$. The set of adjacencies of *B* is $\left \{ X^{h} \bar {X}^{t}, X^{t} \bar {X}^{h} : X \in \mathcal {V}~\text {and degree of}\ X\ \text {is 4}\right \} \cup \left \{ X^{h} X^{t}: X \in \mathcal {V}~ \text {and degree of \textit {X} is 2} \right \}$. The set of adjacencies of *C* is defined adding to *C* an adjacency in $\left \{X^{h} Y^{h}, X^{h} \bar {Y}^{h}, \bar {X}^{h} Y^{h}, \bar {X}^{h} \bar {Y}^{h} \right \}$ for each $XY \in \mathcal {B}$, and an adjacency in $\left \{X^{t} Y^{t}, X^{t} \bar {Y}^{t}, \bar {X}^{t} Y^{t}, \bar {X}^{t} \bar {Y}^{t} \right \}$ for each $XY \in \mathcal {R}$. Figure 4 shows an example of the construction of genomes from a balanced bicoloured graph.

Defining *A,B,C* this way, there is a median *M*⊆*A* ∪ *B* that indicates the number of alternating cycles we have in a maximum edge-disjoint alternating cycle of *G* [29].

As a consequence of *M*⊆*A*∪*B*, we have that *M*∈IG(*A,B*) [19]. So, *M*∈IG(*A,B*) and minimizes *d*_DCJ_(*M,A*)+*d*_DCJ_(*M,B*)+*d*_DCJ_(*M,C*) solving both the DCJ median for this specific instance and the BGD for the general case. It follows, since we can construct genomes *A,B,C* in polynomial time and BGD is NP-hard, that DCJ Intermediate Genome Median is also NP-hard. □

Since the median and consequently the small phylogeny problem are NP-hard also in their intermediate genomes formulation, we propose an approach that combines adjacency weighting methods that are common in adjacency-based algorithms, with the DCJ rearrangement model in the form of intermediate genomes, but without the need to explicitly consider searching for rearrangement events and/or scenarios, which makes the problem much more tractable.

### Maximum weight intermediate genome

#### **Problem 3**

Given genomes *A* and *B* on set of genes $\mathcal {G}$and a set of adjacency weights $W = \left \{w_{ij} \,|\, ij \in \mathcal {G}^{\pm } \times \mathcal {G}^{\pm } \right \}$, find a genome *M* such that

$$ M = \underset{M \in \text{\texttt{IG}}(A,B)}{\arg \max} \sum \delta_{ij}(M) \cdot w_{ij} $$

where *δ*_*ij*_(*M*)=1 if *i* *j*∈*M*, 0 otherwise.

If the genomes *A* and *B* have the same genes, this problem can be solved in polynomial time, since finding a maximum weight set of non-crossing chords in a cycle is equivalent to finding a maximum weight independent set on a circle graph (MWIS) [30]. Therefore, it is possible to find an optimal *M*∈IG(*A,B*) by solving a MWIS for each component of BP(*A,B*).

If *A* and *B* have different gene sets, the problem becomes much harder, since each completion of BP(*A,B*) will give rise to different components and therefore different solutions for the individual MWIS. The naive method of finding the maximum weight IG for all completions is impractical, since, according Eq. (3), there is an exponential number of completions.

A strategy to solve Problem 3 is to search a perfect matching in the graph $\mathcal {H}$ that represents all possible optimal completions in *C* ^∗^, where the weight of each hyperedge is the weight obtained by solving the MWIS for the correspondent component.

Edmonds [31] shows that the maximum weighted perfect 2-matching problem can be solved in polynomial time. It follows directly from the $\mathcal {H}$ representation that

#### **Claim 3**

Suppose that *p*_*AB*_ is even, and ${p_{A}^{o}} \le {p_{A}^{e}}$ and ${p_{B}^{o}} \ge {p_{B}^{e}}$ or ${p_{A}^{o}} \ge {p_{A}^{e}}$ and ${p_{B}^{o}} \le {p_{B}^{e}}$. Then, the Maximum Weight Intermediate Genome problem can be solved polynomially.

Moreover, we have that

#### **Claim 4**

Suppose that *p*_*AB*_ is odd, and ${p_{A}^{o}} \le {p_{A}^{e}}$ and ${p_{B}^{o}} \ge {p_{B}^{e}}$ or ${p_{A}^{o}} \ge {p_{A}^{e}}$ and ${p_{B}^{o}} \le {p_{B}^{e}}$. Then, the Maximum Weight Intermediate Genome problem can also be solved polynomially.

#### *Proof*

Since *p*_*AB*_ is odd, ${p_{A}^{o}} \le {p_{A}^{e}}$ and ${p_{B}^{o}} \ge {p_{B}^{e}}$ or ${p_{A}^{o}} \ge {p_{A}^{e}}$ and ${p_{B}^{o}} \le {p_{B}^{e}}$, there is exactly one hyperedge with 3 elements. The number of hyperedges with 3 elements in $\mathcal {H}$ is limited by ${n \choose 3} = O(n^{3})$. Once one hyperedge with 3 elements is removed, according to Claim 3, finding a perfect 2-matching in the remaining vertices of the graph is polynomial. Therefore, an optimal solution is found in polynomial time by repeating this for all *O*(*n* ^3^) hyperedges with three elements and choosing the solution with maximum weight. □

Unfortunately, the cases where ${p_{A}^{o}} < {p_{A}^{e}}$ and ${p_{B}^{o}} < {p_{B}^{e}}$, or ${p_{A}^{o}} > {p_{A}^{e}}$ and ${p_{B}^{o}} > {p_{B}^{e}}$ are most likely NP-hard, due to the presence of up to *c* (number of chromosomes) triple-matchings in optimal completions, as opposed to just one. This means that the complexity of the Maximum Weight Intermediate Genome problem is still open for the general case. However, considering that the number of chromosomes is constant, we have the following interesting result from the theoretical point of view.

#### **Theorem 3**

There is a polynomial time FPT algorithm for the Maximum Weight Intermediate Genome problem when it is parameterized by the number *c* of chromosomes.

#### *Proof*

Claim 3 and 4 guarantee that there is a polynomial time algorithm if ${p_{A}^{o}} \le {p_{A}^{e}}$ and ${p_{B}^{o}} \ge {p_{B}^{e}}$ or ${p_{A}^{o}} \ge {p_{A}^{e}}$ and ${p_{B}^{o}} \le {p_{B}^{e}}$. If ${p_{A}^{o}} < {p_{A}^{e}}$ and ${p_{B}^{o}} < {p_{B}^{e}}$, or ${p_{A}^{o}} > {p_{A}^{e}}$ and ${p_{B}^{o}} > {p_{B}^{e}}$, using a polynomial algorithm for maximum weighted perfect 2-matching and claim 1, we have a FTP algorithm with parameter *c*. □

## Ancestral reconstruction algorithms

In this section we describe the practical algorithms that were used for our proposed ancestral reconstruction method. First, we discuss how adjacency weights can be obtained. Then, how these weights are used by a heuristic to find candidate intermediate genomes for the ancestral nodes of the input tree.

### Finding adjacency weights

Adjacency weights were obtained using two methods. First, using the software DeClone [32], that randomly samples evolutionary scenarios and assign weights based on how often an adjacency is present on those scenarios. The parsimony score of a given scenario is determined by the number of gains/losses of adjacencies along the branches of the tree. DeClone samples scenarios depending on a parameter *kT*. When *kT* is close to zero, only optimal scenarios (with minimal parsimony score) are sampled, and as *kT* increases, sub-optimal scenarios have a higher chance of being sampled. The weights for each adjacency at each internal node depend on how often this adjacency is observed at this internal node. Typical values include *k* *T*=0.1 for sampling optimal scenarios almost exclusively, and *k* *T*=1 for a more balanced distribution including non-optimal scenarios [18].

We also propose a second way of deriving adjacency weights, inspired by the weighting scheme used in InferCARs [12]. Given a rooted phylogenetic tree *T*, let *w*_*α*_(*i* *j*) denote the weight of adjacency *ij* at a node *α*. Weights in all nodes are recursively defined by

4 $$ w_{\alpha}(ij) = \frac{D_{L} \cdot w_{R}(ij) + D_{R} \cdot w_{L}(ij)}{D_{L} + D_{R}}  $$

where *D*_*L*_ (*D*_*R*_) is the distance to the left (right) child of *α*, and *w*_*L*_(*i* *j*) (*w*_*R*_(*i* *j*)) is the weight of *ij* at the left (right) child of *α*. For leaf nodes, *w*_*α*_(*i* *j*)=1 if the adjacency is present and *w*_*α*_(*i* *j*)=0 otherwise.

**Fig. 5:**
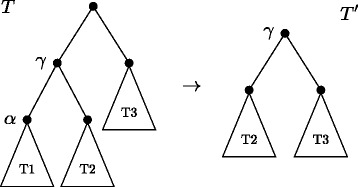
To find adjacency weights for node *α* on a tree *T*, a new tree *T* ^′^ is created where *α* and its subtree *T*1 are removed, and *T* ^′^ is rerooted at *γ*, the parent node of *α*. Then, Eq. (4) is applied to find weights for *γ*, which are then assigned to node *α* on the original tree *T*

To define the weights in our approach, we proceed as follows: for every internal node *α*, let *γ* be the the parent node of *α*, and create a new tree *T* ^′^ by removing from *T* the subtree defined by the node *α*. Then, remove the original root and reroot *T* ^′^ at the node *γ* and use the recurrence equation above to find *w*_*γ*_(*i* *j*) for all adjacencies *ij*. The adjacency weights for *α* are then *w*_*α*_(*i* *j*)=*w*_*γ*_(*i* *j*) for each *ij*. An example is shown on Fig. 5.

The motivation for using this weighting algorithm is that, while reconstructing a particular node *α*, the information from the leaves is given in the form of the breakpoint graph, while the weights that will guide the reconstruction of the intermediate genome should reflect information from the “other side” of the tree. The experimental results show, somewhat surprisingly, that this simple weighting scheme not only is faster than DeClone, but also increases the quality of the reconstruction.

### Estimating branch lengths

For the InferCARs weight algorithm, branch lenghts are needed. Since branch lengths are not always available, we tested how different estimation methods might impact the adjacency weights and consequentely the ancestral reconstruction. For this, we implemented two classic methods of branch length estimation, Minimum Evolution [33] and Fitch-Margoliash Least Squares [34], briefly described in the following.

Let *T* be an unrooted tree with *k* leafs and *n*=2*k*−3 edges, with edge lengths denoted by the vector *w*=(*w*_1_,…,*w*_*n*_). Let *M* be a *m*×*n* matrix, where $m={k \choose 2}$. Each column of *M* represents a branch length, and each row a pairwise comparison between two leafs of *T*. An element *m*_*ij*_ of *M* is 1 if the edge *j* is present in the tree path from the two leafs being compared, and *m*_*ij*_=0 otherwise. Let *d*=(*d*_1_,…,*d*_*m*_) be a vector where each element *d*_*i*_ stores the DCJ-Indel distance of the two genomes being compared on this row *i*. Therefore, for *k*>3 leafs, we have *m*>*n* and *M* *w*=*d* is an over-determined equation system. Then, as proposed by Fitch and Margoliash [34], a good candidate for the edge weights is the vector *w* ^∗^ that minimizes the least squares error, that is,



Another idea is to assume that the pairwise distances in *d* are a lower-bound for the tree traversal distances, and find edge lengths that satisfy this restriction and have minimum total sum. This method, called Minimum Evolution by Waterman et al. [33], is based on solving the following Linear Programming formulation:

$$\begin{array}{*{20}l} \text{minimize } & \; \sum\limits_{i=1}^{n} w_{i} \\ \text{subject to } & \; {Mw} \geq d \\ & \; w_{i} \geq 0, \quad i = 0,\dots,n \end{array} $$

### An algorithm for the IG-Indel small parsimony problem

Given a rooted phylogenetic tree with genomes at the leaves and a set of adjacency weights, our method works in a bottom-up fashion, by choosing two leaves with the same parent, reconstructing the ancestor at this parent node, and labeling this current node as a leaf, until the root of the tree is reconstructed.

At each node being reconstructed, given the two children genomes and a set of adjacency weights, a heuristic for the Maximum Weight Intermediate Genome (MWIG) problem is called, which tries to quickly find an optimal completion with high adjacency weight.

To do that, we build the hypergraph $\mathcal {H}$ representing all optimal completions $\mathcal {C}^{*}$, but ignore triple matchings, focusing only on 2-matchings present in optimal completions, as given by the sets *T*_*i*_, *i*=1,…,7. The weight of an edge in $\mathcal {H}$ is given by the solution of a MWIS on the component correspoding to the given edge. If *p*_*AB*_ is even, there is a perfect matching in $\mathcal {H}$ corresponding to an optimal completion. We find a maximum weight perfect matching using BlossomV [35]. Then, from each MWIS solution for the matched components, we get adjacencies to build a genome *G* that is a high weight solution for the MWIG. If *p*_*AB*_ is odd, we could use Claim 4 strategy of removing every possible triplet of $\mathcal {H}$ and solving the even case as described, picking then the combination with highest weight. Since the number of triplets can be very high, we chose to solve this in a faster way by adding three dummy nodes *v*_*a*_, *v*_*b*_, and *v*_*ab*_ to $\mathcal {H}$, connected with zero weights to all vertices corresponding to *A*-, *B*- and *AB*-paths, respectively, artificially transforming $\mathcal {H}$ in a even *p*_*AB*_ case, and then finding a maximum weight perfect matching on $\mathcal {H}$. The three components that are matched to the dummy nodes are then combined, and a MWIS is solved for this triplet.

A pseudocode of the proposed method, which we call IG_SMALL_PHYLOGENY, is given at Algorithm 1.



## Results

We implemented our algorithms in a software called RINGO (ancestral Reconstruction with INtermediate GenOmes), available at https://github.com/pedrofeijao/RINGO. We created several simulated datasets to test our proposed algorithms and compare with other existing approaches. RINGO was ran with DeClone weights for *k* *T*=0.1, *k* *T*=0.4 and *k* *T*=0.8, and also our custom weight algorithm. For the custom weights, we used the branch lengths given from the simulations, and also tested with branch length estimates given by Minimum Evolution and Least Squares.

We compared RINGO with two other methods for ancestral reconstruction of unequal content genes, MGRA [9] and PMAG + [15], implemented in the tool MLGO [16].

### Simulated datasets

The simulated datasets were created using a similar procedure as in [19], with a few extra parameters to include indel events. A birth-death model with a birth rate of 0.001 and a death rate of 0 generates an ultrametric tree with *N*=12 leaves, and the branch lengths are disturbed by multiplying by *e* ^*d*^, where *d* is a real number uniformly chosen from the interval [−2,+2]. The branch lengths are then rescaled so the tree has a diameter *D*∈{0.5*n*,1*n*,1.5*n*,2*n*,2.5*n*}, where *n*=1000 is the number of genes, and the diameter is the maximum distance between two leaves.

The root node is labeled with an unichromosomal genome with 1000 genes, and evolution is simulated along the edges by performing a number of random events defined by the edge length. Events are chosen randomly between reversals, deletions and insertions, with probability 1−*P*, *P*/2 and *P*/2 respectively, with *P*∈{0,0.2,0.4,0.6}. The length of an indel is sampled uniformly from [1,*I*], with *I*∈{1,5}. Although the expected size of the leaf genomes is 1000, there is not guarantee that genomes will have the same size. For each combination of *D*, *P* and *I*, we generated 20 datasets.

## Discussion

All algorithms were compared in terms of quality of the reconstruction, DCJ distance to the correct ancestral genomes, and running time.

**Fig. 6:**
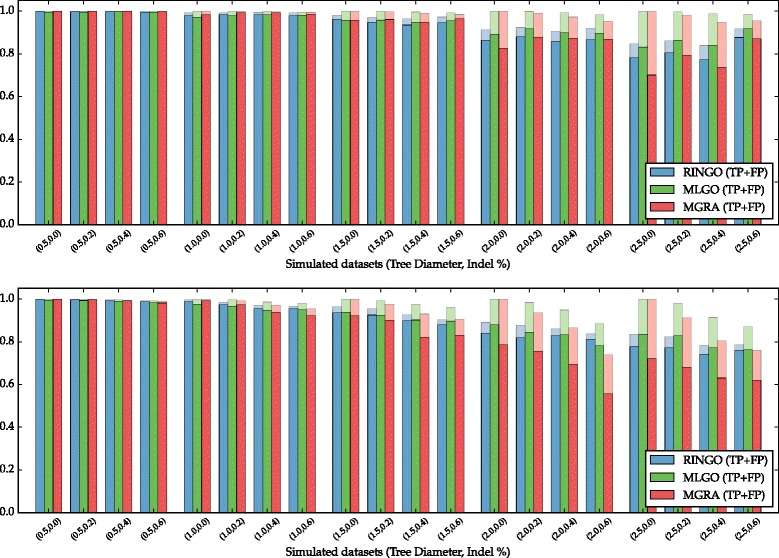
Quality of the adjacency reconstruction for each dataset, with single gene indels (*I*=1, *top plot*) and with indel size ∈[1,5] (*I*=5, *bottom plot*). Each column group represents the average results of RINGO (with custom adjacency weights and branch length estimation with ME), MLGO and MGRA on each dataset, with the percentage of true positives and false positives, when comparing the simulated adjacencies and the reconstructed adjacencies in all internal nodes of the simulated trees

The quality results of all simulations are summarized on Fig. 6. Each column represents the average results of RINGO, MLGO and MGRA on each dataset, showing the average number of true positives and false positives, when comparing the adjacencies of the simulated and the reconstructed genomes, in all internal nodes of a given tree. More detailed results are given on Table 1, that also shows all variations of the RINGO algorithms.

**Table 1:** Average adjacency reconstruction, in terms of true positives (TP) and false positives (FP) for all tested algorithms, for all datasets grouped by tree diameter

Diameter (D)	0.5 *n*		1.0 *n*		1.5 *n*		2.0 *n*		2.5 *n*	
Adjacency results (%)	TP	FP	TP	FP	TP	FP	TP	FP	TP	FP
*Unitary Indels*										
RINGO – Sim. branch lengths	99.8	0.2	99.1	0.7	94.0	3.1	87.9	4.3	81.8	5.5
RINGO – Est. branch lengths with ME	99.8	0.2	99.0	0.7	93.7	3.0	87.7	4.1	80.6	5.6
RINGO – Est. branch lengths with LS	99.8	0.2	99.0	0.7	93.4	3.0	86.7	4.1	80.7	5.4
RINGO – DeClone weights, *k* *T*=0.1	99.6	0.6	98.1	2.0	92.1	7.8	86.9	8.7	80.7	11.0
RINGO – DeClone weights, *k* *T*=0.4	99.6	0.6	98.1	1.9	92.5	9.1	88.4	9.9	82.7	16.8
RINGO – DeClone weights, *k* *T*=0.8	99.2	1.2	97.5	2.9	91.8	9.9	88.0	10.5	82.5	17.1
MLGO	99.6	0.3	98.6	1.3	94.6	5.0	91.8	7.8	85.6	13.8
MGRA	99.9	0.0	99.3	0.5	95.1	3.5	85.8	12.8	70.4	26.7
*Indel length* ∈[1,5]										
RINGO – Sim. branch lengths	99.4	0.3	96.7	1.4	92.0	2.6	73.8	6.2	81.0	4.9
RINGO – Est. branch lengths with ME	99.4	0.3	96.6	1.4	91.6	2.6	71.8	5.8	79.5	4.3
RINGO – Est. branch lenghts with LS	99.4	0.2	96.7	1.4	91.4	2.6	70.2	5.1	77.0	3.8
RINGO – DeClone weights, *k* *T*=0.1	99.3	0.7	95.4	5.7	90.6	10.4	72.0	14.1	79.3	13.4
RINGO – DeClone weights, *k* *T*=0.4	99.3	0.7	95.7	5.7	91.3	11.0	75.2	19.9	82.1	16.9
RINGO – DeClone weights, *k* *T*=0.8	99.1	1.1	95.1	6.8	90.7	12.3	74.9	20.3	81.9	17.4
MLGO	98.9	0.7	95.9	3.7	90.6	7.0	75.0	15.9	81.7	13.8
MGRA	99.3	0.3	99.7	0.3	94.8	3.8	65.5	34.5	57.4	42.4

RINGO algorithm was tested with the InferCARs adjacency weights, using the simulated tree branch lengths and with branch lenghts estimated with Minimum Evolution or Least Squares methods. RINGO was also ran with DeClone weights, with varying *kT* values

In datasets with small amount of evolution (*D*=0.5 and *D*=1), specially with unitary indels (*I*=1), MGRA has a slightly better quality than the two others. But, as soon as the rearrangement rate increases, MGRA quality decreases rapidly, while RINGO and MLGO quality seems to decrease in a slower, somewhat linear rate.

At higher rates (*D*>1), MLGO has a slightly higher number of true positives, but at the cost of a much higher number of false positives. RINGO is a more conservative method, with the smallest number of false positives in all datasets.

When comparing the datasets with *I*=1 versus *I*=5, we notice a decrease in quality for all algorithms for the larger indels, but MGRA has a slightly larger loss of quality, specially at higher rates of evolution. In fact, in most datasets with *I*=1, increasing the indel probability *P* also increases the quality of MGRA, while the opposite happens for *I*=5. We believe that this might be a consequence of the way that MGRA models the prosthetic chromosomes by adding an edge *v* ^*t*^,*v* ^*h*^ for each missing gene, which implicitly assumes that this gene is part of an unitary indel. In that respect, using a DCJ indel model such as the one in RINGO, that allows for block indels, will give better results when block indels do occur, which we believe is the more realistic case.

**Fig. 7:**
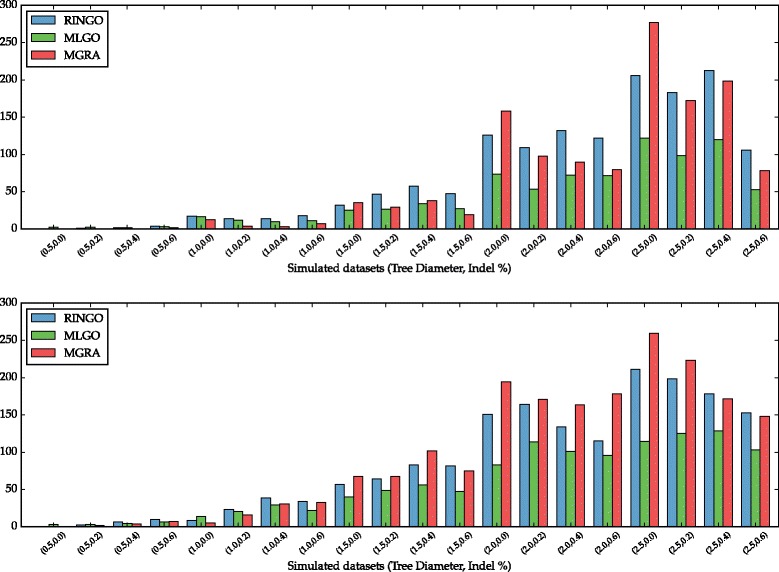
DCJ-Indel distance between the simulated and reconstructed genomes, with only single gene indels (*I*=1, *top plot*) and with indel size ∈[1,5] (*I*=5, *bottom plot*). Each column group represents the average distances of RINGO, MLGO and MGRA on each dataset

Using the DCJ-Indel distance [20], we also measured how far the reconstructed genomes are from the simulated genomes in average, and the results are shown on Fig. 7. As the quality results indicate, at lower rate, specially with unitary indels, MGRA has the smallest distances, but they increase rapidly for higher rates. Comparing MLGO and RINGO for the higher rates, even though MLGO has a higher percentage of false positives, it has the smallest distances to the ancestral genomes. We believe that this is caused by the fact that the DCJ distance strongly penalizes fragmentation. Therefore, comparing conservative methods like RINGO, that have a lower percentage of false positives, with more aggressive methods like MLGO, with more true positives at the cost of higher false positive percentage, the latter methods will have smaller distances. For instance, consider an ancestral unichromosomal genome *A*=(*a,b,c,d*), where the letters represent four blocks. If a method correctly reconstructs all four blocks, but not the connection between them, that is, a fragmented genome *B*=(*a*)(*b*)(*c*)(*d*) with four chromosomes, then we have that the DCJ distance is *d*(*A,B*)=3. Now, consider another method that also reconstructs the four blocks correctly, but gives a wrong ordering, such as *C*=(*a,c,b,d*). Surprisingly, we have that *d*(*A,C*)=2, even though this reconstruction has the same number of correct adjacencies as the previous one, but more false positives. Indeed, for the general case of an ancestral genome *A*=(*a*_1_,*a*_2_,…,*a*_*n*_) and a fragmented reconstruction *B*=(*a*_1_)…(*a*_*n*_), we have *d*(*A,B*)=*n*−1, which is in fact the DCJ diameter for *n* blocks, that is, the maximum possible DCJ distance. Any ordering of the blocks *a*_1_,…,*a*_*n*_, even completely random, will have an equal or smaller distance to genome *A*. Therefore, aggressive methods that try to minimize fragmentation by adding adjacencies, even with small support, will have a smaller distance to the correct ancestral genome, but we argue that this is no indication of a better reconstruction.

While comparing running times, for RINGO and MGRA the determining parameter is the rate of evolution, controlled by the parameter *D*. For MLGO, the running times stayed around one minute regardless of the rate. On Table 2, we show the average running times for all repetitions and indel rates *I*∈{0,0.2,0.4,0.6}, in each of the different diameters, for the two datasets *I*=1 and *I*=5. The running time of RINGO is smaller than MLGO and increases in a much slower rate than MGRA, which increases exponentially for larger rates of evolution.

**Table 2:** Average running time of 20 runs of each algorithm and all indel rates *I*∈{0,0.2,0.4,0.6}, for different tree diameters with two different parameters *I* determining the size of the indels in number of genes

Dataset	*I*=1, unitary indels					*I*=5, indels with 1 to 5 genes				
Diameter (D)	0.5 *n*	1 *n*	1.5 *n*	2 *n*	2.5 *n*	0.5 *n*	1 *n*	1.5 *n*	2 *n*	2.5 *n*
RINGO	3 s	3 s	5 s	7 s	7 s	3 s	4 s	5 s	7 s	8 s
MLGO	1 m 6 s	1 m 10 s	1 m 7 s	1 m 9 s	1 m 16 s	57 s	60 s	1 m 4 s	1 m 7 s	1 m 10 s
MGRA	7 s	1 m 46 s	12 m 12 s	56 m 55 s	2 h 2 m 41 s	23 s	6 m 56 s	48 m 42 s	2 h 1 m 44 s	2 h 40 m 18 s

In summary, these results show that algorithms based on intermediate genomes can perform at quality levels equal or higher than current approaches for ancestral reconstruction, while also being much faster.

## Conclusion

In this paper we proposed a new method for ancestral reconstruction of gene orders for genomes with unequal gene content by expanding a previous approach for genomes with same gene content. The IG algorithm is faster and in many datasets has a better reconstruction quality than MGRA and MLGO, specially for higher rates of evolution. We believe that one of the strongest points of our approach is the use of extra information, in the form of intermediate genomes, and not simply relying on the parsimonious idea of minimizing tree distances. With that, not only the quality of the reconstructed genomes is improved but also the search space is drastically reduced, resulting in faster algorithms. We also think that a combined approach with ideas from both worlds could deliver very good results. As an example, we could think of combining the space reduction power of intermediate genomes with the strong space search techniques of MGRA.

There are many ways that we can improve the ideas presented in this paper. For one, instead of using a heuristic for solving the maximum weight intermediate genome, we will test how solving this problem exactly changes the results, whether by using a FPT such as the one described, or resorting to an integer linear programming for the more complex cases. We also plan to extend the current framework to allow the presence of duplicated genes.

The proposed algorithms were implemented as Python 2.7 scripts in a software called RINGO, that can be downloaded at https://github.com/pedrofeijao/RINGO. Also included are scripts to generate simulations and parse the reconstruction results on the simulated datasets, comparing RINGO with other algorithms.
